# Low-dosage ozonation in gas-phase biofilter promotes community diversity and robustness

**DOI:** 10.1186/s40168-020-00944-4

**Published:** 2021-01-12

**Authors:** Marvin Yeung, Prakit Saingam, Yang Xu, Jinying Xi

**Affiliations:** 1 Environmental Simulation and Pollution Control State Key Joint Laboratory, School of Environment, Tsinghua University, Beijing, 100084 China; 2 Department of Civil and Environmental Engineering, University of Hawaiʻi at Mānoa, 2500 Campus Rd, Honolulu, HI 96822 USA

## Abstract

**Background:**

The ozonation of biofilters is known to alleviate clogging and pressure drop issues while maintaining removal performances in biofiltration systems treating gaseous volatile organic compounds (VOCs). The effects of ozone on the biofilter microbiome in terms of biodiversity, community structure, metabolic abilities, and dominant taxa correlated with performance remain largely unknown.

**Methods:**

This study investigated two biofilters treating high-concentration toluene operating in parallel, with one acting as control and the other exposed to low-dosage (200 mg/m^3^) ozonation. The microbial community diversity, metabolic rates of different carbon sources, functional predictions, and microbial co-occurrence networks of both communities were examined.

**Results:**

Consistently higher biodiversity of over 30% was observed in the microbiome after ozonation, with increased overall metabolic abilities for amino acids and carboxylic acids. The relative abundance of species with reported stress-tolerant and biofilm-forming abilities significantly increased, with a consortium of changes in predicted biological pathways, including shifts in degradation pathways of intermediate compounds, while the correlation of top ASVs and genus with performance indicators showed diversifications in microbiota responsible for toluene degradation. A co-occurrence network of the community showed a decrease in average path distance and average betweenness with ozonation.

**Conclusion:**

Major degrading species highly correlated with performance shifted after ozonation. Increases in microbial biodiversity, coupled with improvements in metabolizing performances of multiple carbon sources including organic acids could explain the consistent performance commonly seen in the ozonation of biofilters despite the decrease in biomass, while avoiding acid buildup in long-term operation. The increased presence of stress-tolerant microbes in the microbiome coupled with the decentralization of the co-occurrence network suggest that ozonation could not only ameliorate clogging issues but also provide a microbiome more robust to loading shock seen in full-scale biofilters.

**Video abstract**

**Supplementary Information:**

The online version contains supplementary material available at 10.1186/s40168-020-00944-4.

## Introduction

Biofiltration is a technology widely applied in the abatement of a wide range of gaseous pollutants [1]. Biofilters are known for their low cost [2–4] and minimal secondary pollution [5, 6]. A persistent issue present in the application of biofilters is the excessive growth of biomass, resulting in clogging, increased pressure drop, and decreased removal performance [7, 8]. Various biomass control methods have been developed to solve clogging issues [9]. One technique of ozone injection was previously reported [10], complemented by another study examining the metabolic activities of the microbiome in biofilters under O_3_ exposure, which concluded that O_3_ exposure led to an increase in the metabolism of numerous carbon sources of lower biodegradability, such as γ-hydroxybutyric acid, d-galactonic acid γ-lactone, d-mannitol, d-cellobiose, and γ-methyl-d-glucoside [11]. Traditionally, O_3_ is regarded as a strong oxidant that purges microorganisms and lowers overall biological activity [12, 13]. However, the opposite response was found in low-dosage O_3_ exposure. For example, low concentrations of O_3_ (e.g., 120 mg/m^3^) were found to improve metabolic activity [14]. It was shown that despite the decrease in biomass, microbial activity for the metabolism of multiple carbon sources increased in the biofilter, implying an inherent change at the microbial level. It is well-known that the microbiome of a biofilter is crucial for effective pollutant abatement [15], yet the exact microbiome and functional changes that occur allowing for increased microbial activity while maintaining system performance with a decrease in biomass remain intriguing and unknown. In this study, 16S rRNA sequences of the v4 region in a controlled biofilter and ozonated biofilter operated in parallel were sequenced in order to investigate the microbial changes leading to the adaptation and performance changes of the microbiome. Using statistical analysis techniques, we attempt to explain the relevance of dominant taxa treating toluene after addition of O_3_. Using the latest databases, we predicted changes in functional and phenotypic characteristics under ozonation and quantified the relationship between microbiome change and removal performance.

## Methodology

### Experimental setup

Two lab-scale biofilters, BF1 and BF2, were constructed as acrylic cylinders with a 12-cm inner diameter and 25-cm height. Each biofilter was packed with porous perlite (0.54 void fraction) to form a 1.6-L filter bed with a height of 15.0 cm. An air compressor (Hailea ACO-318, Fuzhou, China) was used to feed air into the system. The gas flow rate was controlled by a flow meter (Zenxing LZD-4WB, Xianghu, China), leading into a sealed glass bottle containing liquid toluene to form mixed gas. A stainless-steel reactor equipped with a UV lamp (Cnlight ZW23D15W-Z436, Shenyang, China) was installed between the flow meter and the bottle containing liquid toluene, operating at 185 nm to produce gaseous ozone for BF1. For each biofilter, the packing media was initially mixed with 1.0 L of activated sludge collected from a municipal wastewater treatment plant (Xiaojiahe WWTP, Beijing, China). A nutrient solution containing NaNO_3_ (20 g/L), Na_2_HPO (1.6 g/L), and KH_2_PO_4_ (1.04 g/L) was sprayed directly on the filter beds of the two biofilters for sufficient humidity and nutrients. The leachate was discharged every day. The two biofilters were operated in parallel for 160 days in total. Both were operated in identical conditions without ozone for the first 44 days, and BF1 was fed with 200 mg/m^3^ gaseous ozone after day 45.

### Performance analysis methods

Gaseous toluene concentrations were measured using a gas chromatograph (SHIMADZU, GC-14C, Kyoto, Japan) with a flame ionization detector (GC/FID). The temperatures of the column, injector, and detector were set at 100, 150, and 150 °C, respectively. The CO_2_ level in the mixed gas sample was measured with an infrared detector (Testo, Testo 535 CO_2_, Lenzkirch, Germany), and ozone concentration was monitored by a UV absorbance detector (2B Technologies Inc, 106-M, Boulder, USA).

All concentrations were measured daily at 9:00, 13:30, and 18:00 (GMT +8) with six replicates at each point. The highest and lowest replicate points were discarded, and the remaining four data replicates were averaged to yield three resulting data points for each day. Removal efficiency and mineralization rate data used in correlation analysis with the microbiome at a particular time point were obtained by averaging the performance data of 3 days surrounding the date of microbial sampling, including the day of sampling and 1 day before and after.

### Microbial sampling

Microbial samples were taken from both biofilters at days 38, 52, 66, 80, 94, and 160. Packing media were taken from depths of 1, 7, and 15 cm of the filter bed, and the biofilms were detached and suspended in phosphate buffer saline (PBS) by sonication at 425 W, 21–25 kHz for 10 min (Ningbo Science Biotechnology SCIENTZ-IID, Ningbo, China). The sonicated suspension was centrifuged at 10000×*g* for 1 min and resuspended in 5 ml PBS. To exclude dead cells within the community, a fluorescent dye (propidium monoazide, PMA) was used to treat the microbial suspension by inactivating the DNA of cells with damaged cell membranes as well as exposed DNA [16]. PMA (Biotum, PMA™ dye, Fremont, USA) stock was prepared by dissolving 1 mg PMA in 100 μL of 20% dimethyl sulfoxide (DMSO) and stored at − 20 °C; 2.5 μL of 20 mmol/L PMA solution was added into a 500 μL microbial suspension. The mixture was incubated at room temperature for 5 min and mixed every 60 s. The tubes were placed horizontally on ice and exposed to a 650 W halogen light at a 20-cm distance for 4 min. Then, DNA from PMA-treated aliquots were isolated with the FastDNA® SPIN Kit for Soil (MP Biomedicals, Solon, USA) following the manufacturer’s instructions.

Samples from days 66, 80, 94, and 160 were divided into three identical samples after PMA treatment and before DNA extraction; therefore, each sample was represented in triplicate to counter systematic biases from DNA isolation, PCR, and sequencing procedures. Samples from days 38 and 52 were not sequenced as triplicates and therefore were not included in all analyses of this study to ensure consistent biological and statistical validity. They were used only for verification of factual differences caused by ozone and not systematic errors, as seen in Supplementary Material 1.

### High-throughput sequencing and data analysis

The primer 515F (5′-GTGCCAGCMGCCGCGGTAA-3′) and reverse primer 806R (5′-GGACTACHVGGGTWTCTAAT-3′) were used with 12 bp barcode (Invitrogen, Carlsbad, CA, USA) [17]. PCR reactions, containing 25 μl 2x Premix Taq (Takara Biotechnology, Dalian, China), 1 μl of each primer (10 mM), and 3 μl DNA (20 ng/μl) template in a volume of 50 μl, were amplified by thermocycling: 95 °C for 3 min, followed by 30 cycles of 98 °C for 20 s, 55 °C for 15 s, and 72 °C for 15 s, with a final extension at 72 °C for 10 min. The PCR instrument used was a BioRad S1000 (Bio-Rad Laboratory, Irvine, USA). The length and concentration of the PCR product were detected by 1% agarose gel electrophoresis. Samples with bright main strips between 288 and 310 bp were used for further experiments. PCR products were mixed in equidensity ratios according to the GeneTools analysis software (Version4.03.05.0, SynGene). Then, the mixture of PCR products was purified with an EZNA gel extraction kit (Omega, Norcross, USA). Sequencing of the 16S rDNA V4 region was carried out on an Illumina Miseq platform. Barcodes and adapter sequences were trimmed with Cutadapt [18], then truncated at 210 bp and denoised with DADA2 to formulate the ASV (amplicon sequence variants) table [19]. Taxonomy of 16S rRNA sequences were classified with the Silva 16s rRNA database (release 138) at 99% similarity with the Naïve-Bayes algorithm for all analyses requiring taxonomy [20, 21]. The software Bugbase was used for functional prediction, which required the Greengenes database, for which Greengenes (version 13_5) was used [22]. Function predictions were done using Bugbase, along with PICRUSt2 for the prediction of MetaCyc pathways(version 2.1.2b) [23–25]. Statistical analyses, including alpha and beta analysis, were conducted in the R package Phyloseq (version 1.3) [26]. The R package ALDEx2 (version 1.2) was used to statistically identify pathways highly specific to ozone and control biofilters [27]. Correlation analyses were performed and plotted using Pearson correlation incorporated in the R package ggcor [28]. Co-occurrence network analyses were conducted with the molecular ecological network analyses with Spearman correlation, Bray-Curtis dissimilarity, and a RMT (random matrix theory) threshold of 0.81 [5]. Networks were plotted with the Java software Gephi [29]. All R packages were conducted under R version 3.6. LEfSe analysis was performed on the only galaxy platform maintained by the author, with *p* value threshold of the Wilcoxon test set to 0.05 and LDA log-score threshold set to 3.0 [30].

### Metabolic activity analysis

The suspension from sonification containing microbiomes detached from the packing media as described in the “Microbial sampling” section was diluted in PBS to obtain the optical density at 600-nm wavelength (O.D._600_) at 0.05. The ECO plate (Biolog, Inc, Hayward, USA), with 31 various sources of carbon substrates mixed with tetrazolium dye, was prepared for the determination. Then, 150 μL of the microbial dilutions was inoculated to each well of the ECO plate and incubated at 30 °C. The plate was observed for the absorbance at 600 nm regularly over a period of 3 days by the microplate reader (Molecular devices, Spectramax M5, San Jose, USA). The absorbance over time from wells containing carbon sources from the same group (e.g., amino acids) was averaged, and the absorbance from the control well was deducted to avoid systematic error and obtain the average metabolic rates of different groups of carbon sources.

## Results

### Sequencing results

A total of 9347–59,720 sequences of 210 bp were obtained. Samples were rarefied at 13,000 sequences, resulting in 24 samples. A total of 3295 ASVs were identified after denoising.

### System performance

The effects of O_3_ on performance data during the period was reported in detail in a previous publication [11], in which both biofilters had a removal rate of approximately 65–70% when treating inlet toluene ranging from 500 to 1500 mg/m^3^, with no significant difference under *t* test (*p* = 0.62).

### Biodiversity

**Fig. 1:**
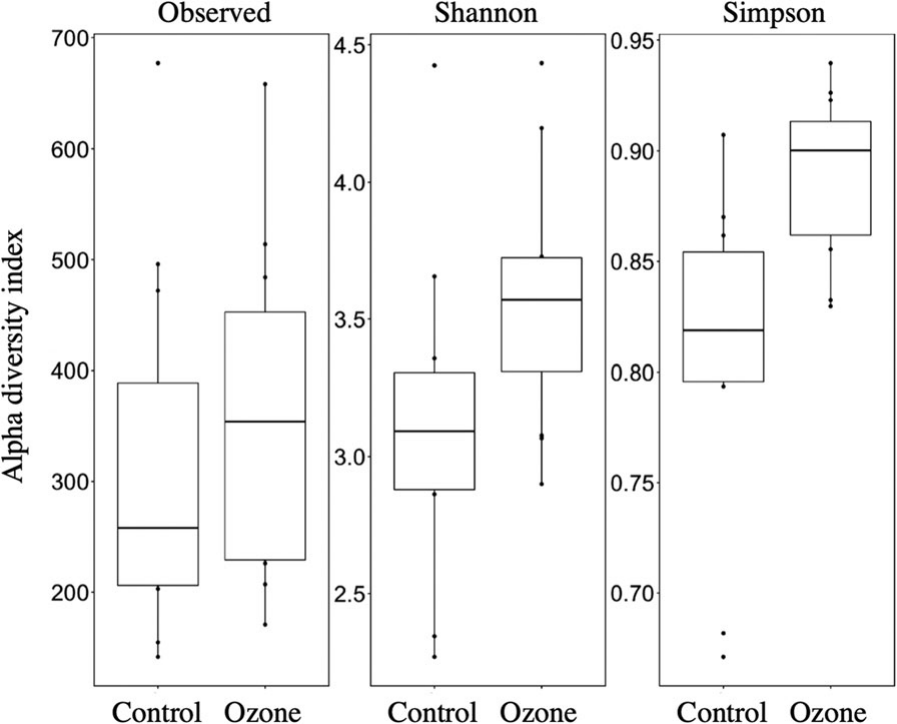
Observed, Shannon, and Simpson diversity indices of samples of the two biofilters

Biodiversity indices comparing the two biofilters are shown in Fig. 1. The observed intra-group diversity spanned over 400 ASVs in both groups, but the median and average diversity of the ozone biofilter were consistently higher than those of the control samples, indicating an increase in phylogenetic diversity range and average evenness.

### Community differences and propensity of variables

**Fig. 2:**
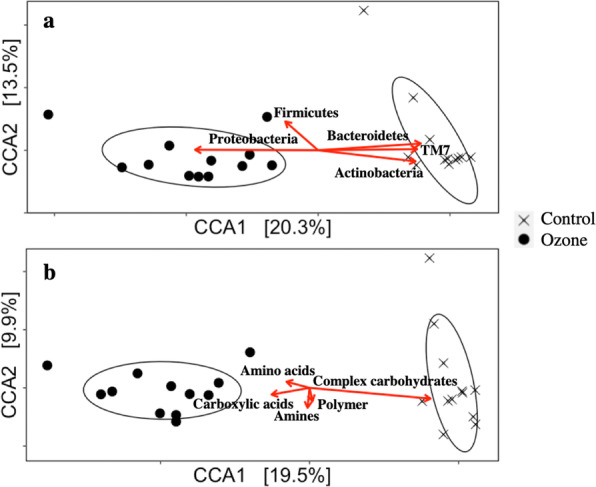
**a** CCA analysis of ASV composition with relative abundance of *Actinobacteria*, *Proteobacteria*, *Firmicutes*, *Bacteroidetes*, and *TM7* as constraining variables. **b** CCA analysis of ASV composition with the metabolic rate of amino acids, carboxylic acids, amines, polymers, and complex carbohydrates as constraining variables

A clear separation of microbiome composition was seen between the two biofilters. As the intra-group differences also fluctuated considerably with time, the relative abundances of major phyla *Actinobacteria*, *Proteobacteria*, *Firmicutes*, *Bacteroidetes*, and *TM7* were added as constraining variables in Fig. 2a to show the overall compositional differences in ASV levels and the abundance differences of major phyla. Detailed bar plots showing the relative abundances at all time points at the phylum and genus levels are provided in Supplementary Material 2. In addition to the prediction of microbiome functions, microbiome phenotypic metabolizing activity from Biolog ECO plates was adopted as constraining variables to show the preference of communities for different carbon sources in Fig. 2b. *Proteobacteria* remained dominant in relative abundance after ozonation; *Firmicutes* were increased after ozonation, consisting mostly of gram-positive species; and *Actinobacteria*, another phylum with mostly gram-positive species, were suppressed after ozonation. The metabolization ability of groups of amino acids and carboxylic acids increased in the microbiome under ozonation, each containing a consortium of numerous compounds from the groups, while metabolization rates for complex carbohydrates decreased. The metabolization data of individual compounds are provided in Supplementary Material 3.

### Functional characteristics and traits caused by ozonation

**Fig. 3:**
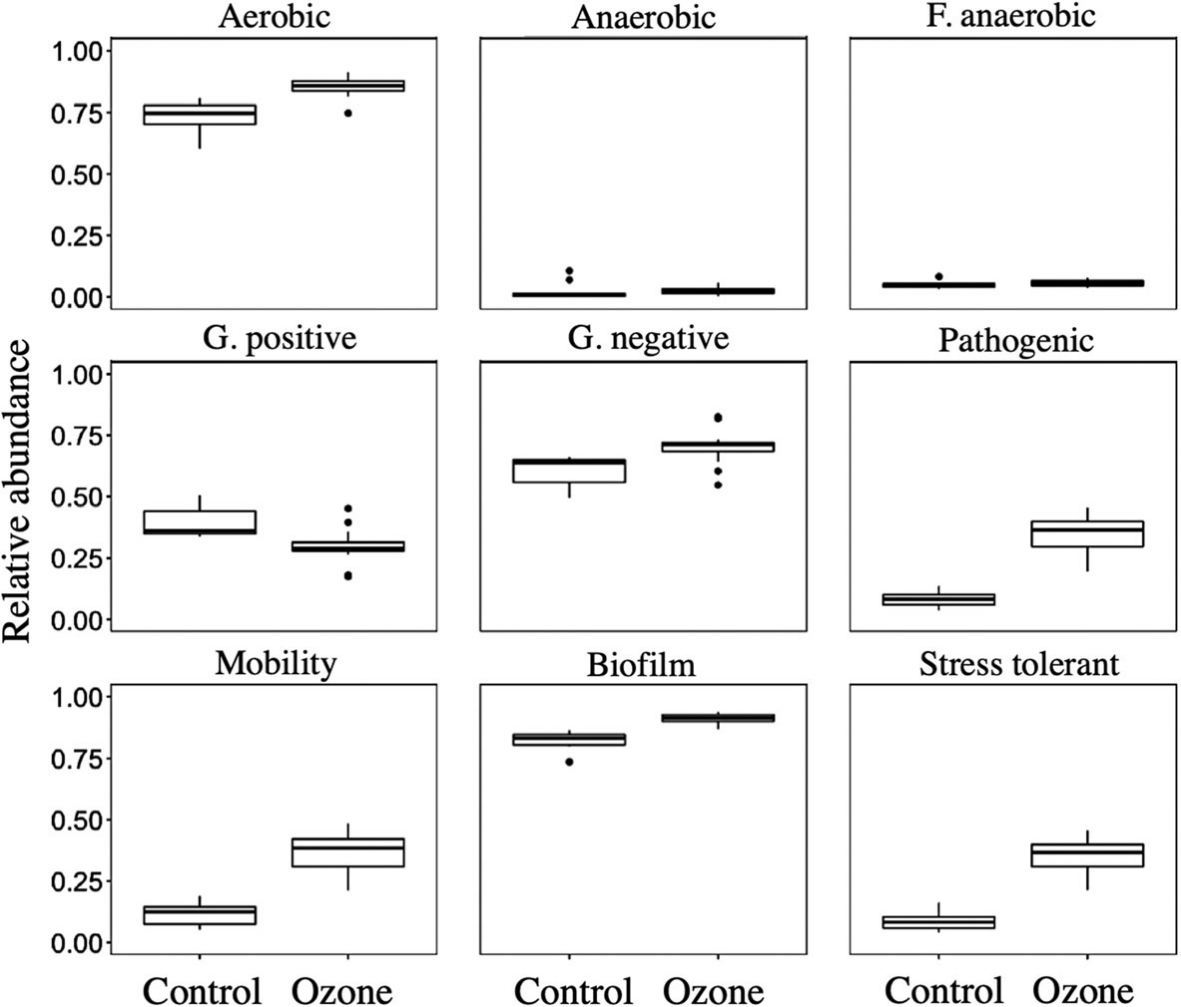
Community functional mapping using the Bugbase database, for quantifying relative abundance of traits in nine categories

The phenotypic traits of the microbiome from both biofilters are shown in Fig. 3. Increases in aerobic, gram-negative, pathogenic, mobility, biofilm-forming, and stress-tolerant species were seen after ozonation, while proportion of gram-positive species decreased.

**Fig. 4:**
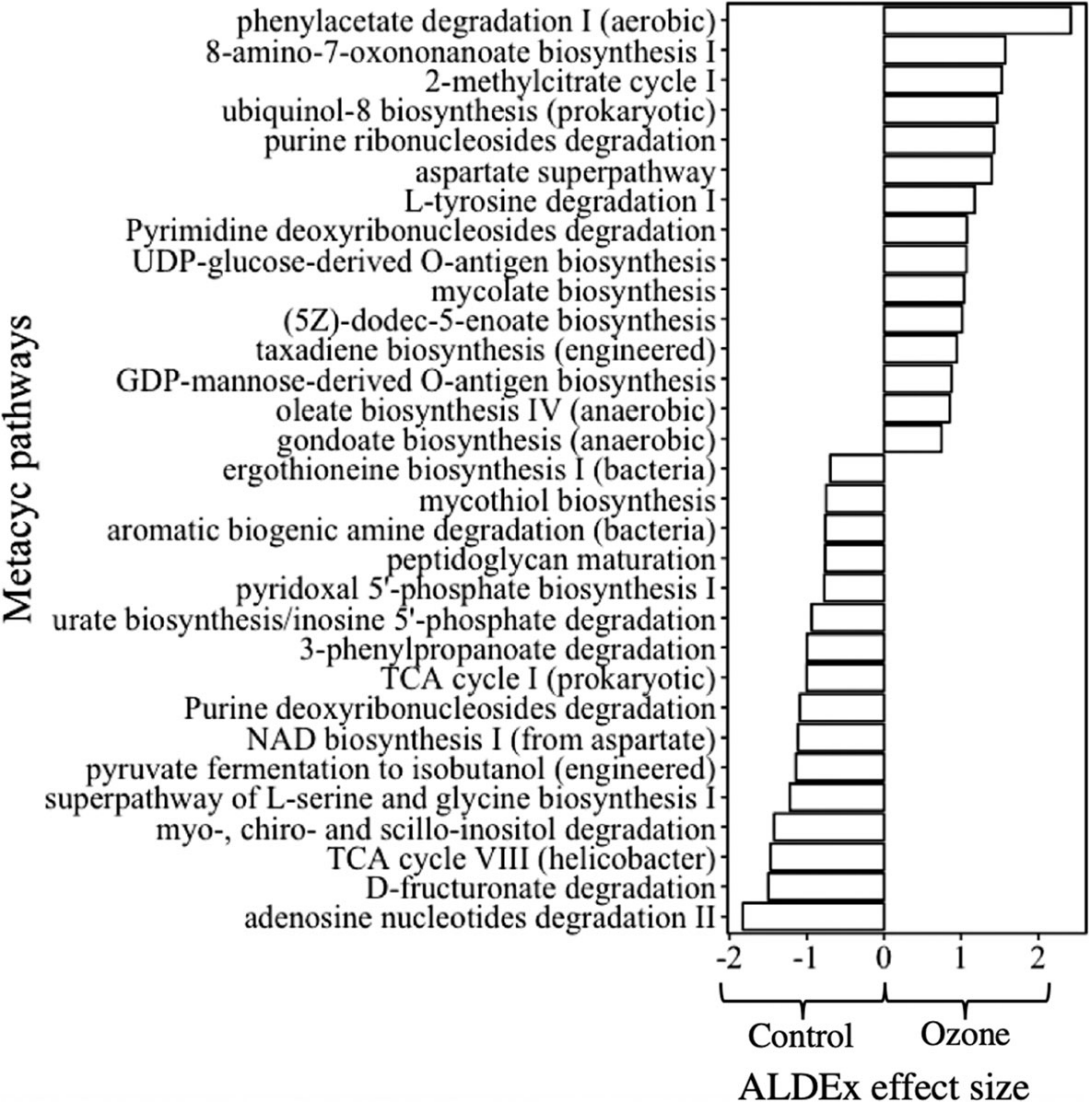
Statistically differentiated MetaCyc pathways in the two biofilters predicted by PICRUSt2 and filtered with ALDEx2 corrected Wilcoxon test

MetaCyc pathways predicted by PICRUSt2 that changed substantially with an ALDEx effect size of over 2.5 are shown in Fig. 4. Among 15 pathways highly specific to ozonation, mycolate biosynthesis, taxadiene biosynthesis (engineered), the superpathway of heme biosynthesis from glycine, and (5Z)-dodec-5-enoate biosynthesis are crucial pathways for the assembling of proteins for cell membrane synthesis. Although gram-negative bacteria increased with ozonation, the repair and synthesis of cell membranes to mitigate oxidative disruption due to ozone was seen in the microbiome, along with the production of antioxidants such as heme and UDP-glucose-derived O-antigen biosynthesis. Of all enriched pathways after ozonation, the phenylacetate degradation I (aerobic) pathway was the most significant with an effect size of over 2.5. Phenylacetate and the degradation of other compounds containing acetate groups are widely seen in the essential steps of toluene and xylene degradation, two recalcitrant compounds commonly seen as VOCs pollutants [31, 32].

A decrease in pathways from the TCA cycle was seen in ozonation filter, and pathways for the degradation of other metabolites in VOCs degradation such as 3-phenylpropanoate and inositol increased after ozonation, indicating a variety of nonuniform changes in pathways. No decisive changes affecting the overall functional degrading ability were seen between groups, but rather a mixture of pathway changes, most of which were specific to particular metabolites before complete mineralization.

### Correlation of system performance and dominant taxa

**Fig. 5:**
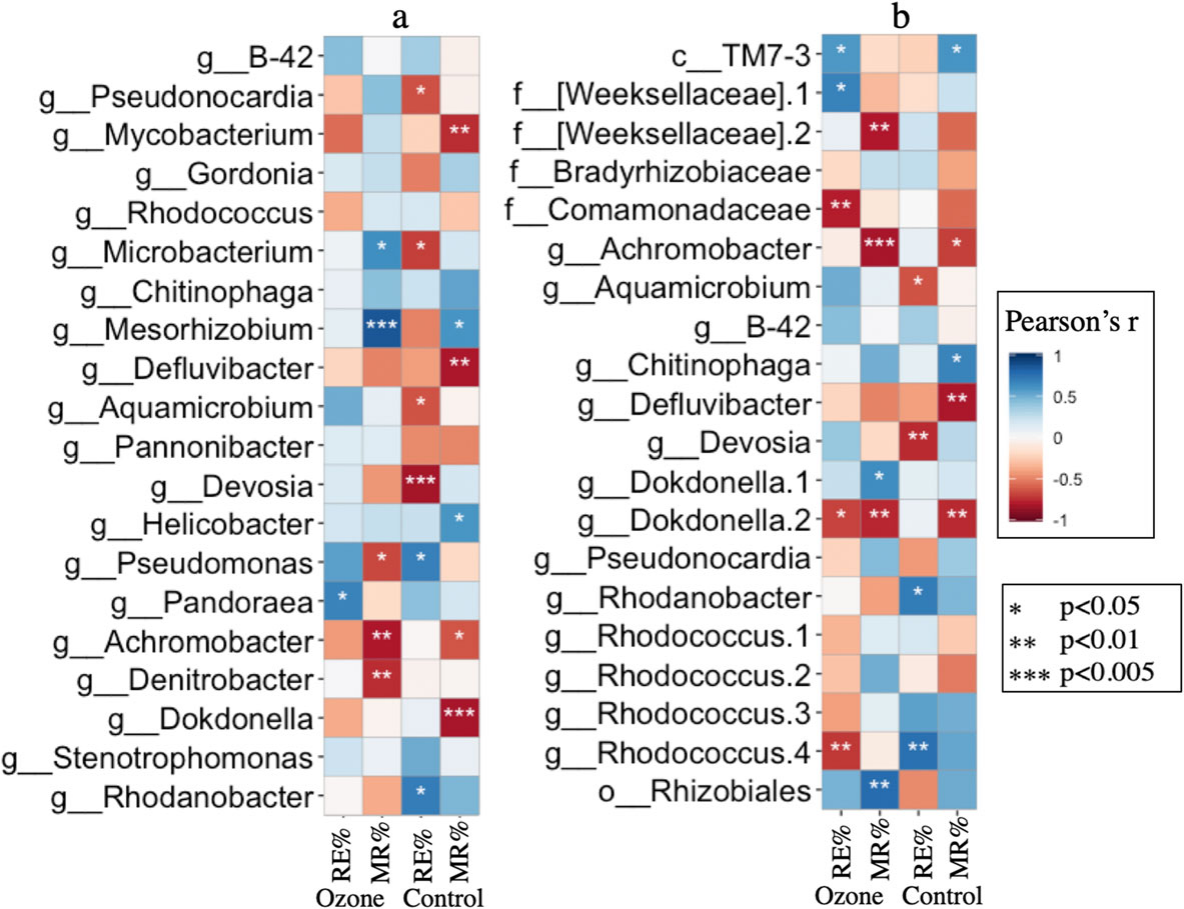
Top 20 taxa in average relative abundance in all samples and their Pearson correlation with removal efficiency (RE%) and mineralization rate (MR%) in two biofilters. **a** Genus level. **b** ASV level, named for the lowest taxa rank identified with confidence over 97%, numbers are appended after names if different ASVs share the same lowest rank name

Due to the high functional redundancy known in bacterial populations, the majority of functions are commonly assumed to be performed by the most abundant taxa in a certain microbiome [33]. The dominant taxa on both ASV and genus level with top 20 relative abundance were selected, and their abundance in different samples and operation time were plotted with the removal efficiency and mineralization rates of corresponding samples in order to investigate differences and similarities in the reactions of dominant taxa and their relationship with performance measures, as shown in Fig. 5. By comparing both removal efficiency and mineralization rate, insights can be drawn from the removal of toluene and the complete degradation to CO_2_ and hence intermediate metabolites.

Both the correlation of ASV and genus level phenylacetate were chosen to investigate possible intra-genus differentiation and radicality of differences. Nine out of 20 genera were always correlated with removal efficiency in both ozone and control biofilter, whereas only 3 out of 20 ASVs were always correlated with removal efficiency, indicating a high specificity and different reaction of ASVs in the same genus toward ozonation. All four ASVs from the genus Rhodococcus present in this system were strongly proportional with removal efficiency in the control biofilter but negatively correlated with removal efficiency in the ozonated biofilter.

### Topological analysis and co-occurrence network construction of microbiome

**Fig. 6:**
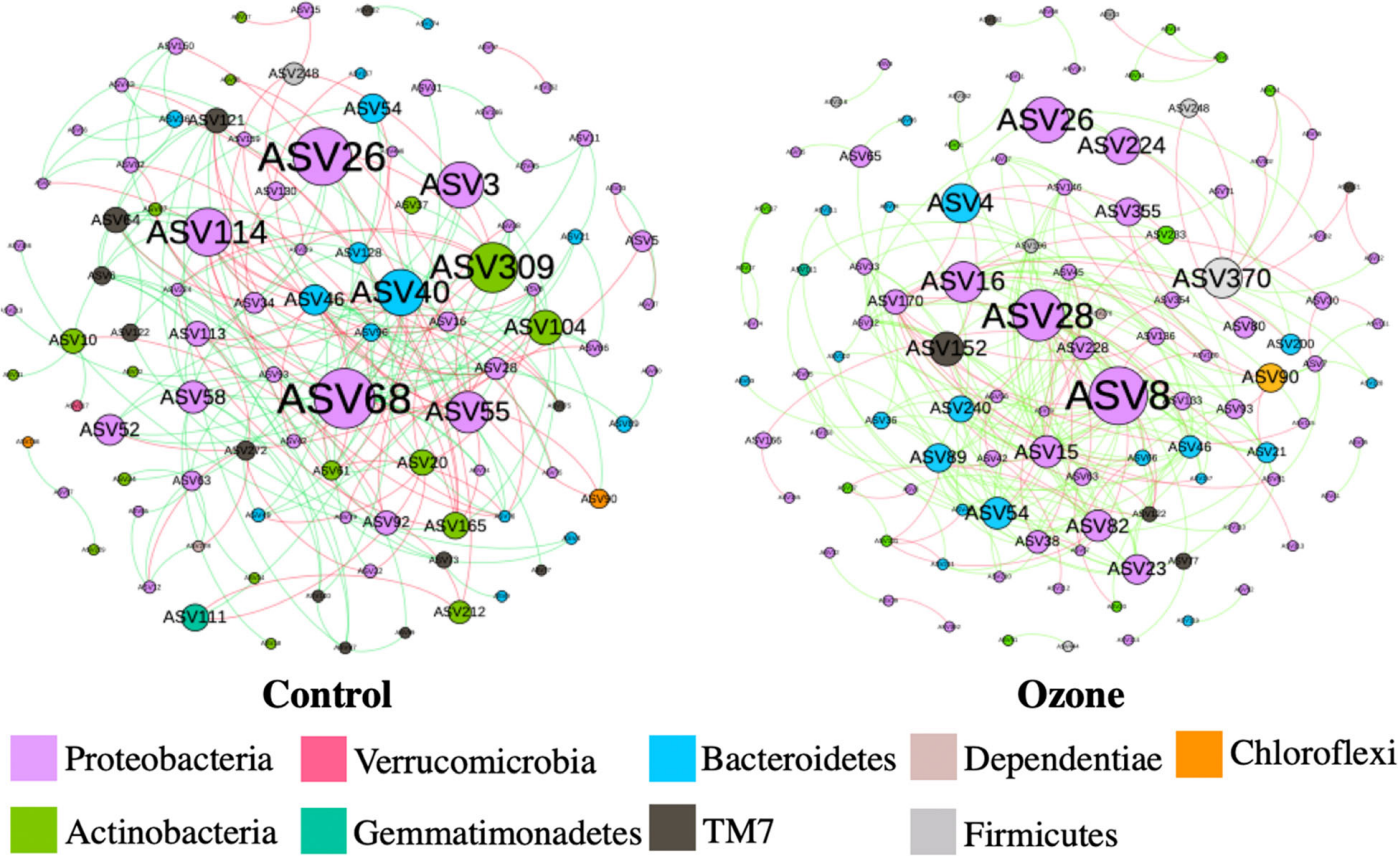
Microbial network in two biofilters with node color as phylum, node size for betweennes, and edge color as positive or negative correlations

Two co-occurrence networks were constructed with 91 and 107 nodes, 146 and 256 links, and 5.1 and 3.6 average path distances for the control and ozone biofilters, respectively, with the increase of average degree from 3.2 to 4.7, respectively, under identical specifications for network construction, as seen in Fig. 6. A more connected and even network was seen in the ozone microbiome; a network with shorter path distances coupled with smaller centralization of betweenness is commonly seen as more stable, as the major hubs are more diverged and less nodes are likely to be affected by shocks. Major hubs in the control biofilter such as ASV68 and ASV309 were substantially irrelevant in the ozone biofilter, indicating a radical change in the microbial network and hub distribution, yet ASV26, corresponding to the genus *Pandoreae*, remained highly relevant in betweenness and degree count in both systems. Exact ASV taxonomic assignments can be seen in Supplementary Material 4.

## Discussion

Pollutant removal performance is the main goal of improvement attempts with regards to alleviating clogging issues. Numerous studies have reported that ozonation improves or maintains VOCs’ removal performance while significantly reducing biomass growth rates [14, 34, 35]. The results from our study, conducted for a period of 160 days, were consistent with those of previous studies in that no statistically significant changes were seen in terms of removal efficiency when operating at a relatively higher loading rate (fluctuating between 20 and 70 mg/L/h) compared to usual full-scale applications, showing ozonation to be a suitable candidate for full-scale application with the ease of retrofitting and flexible manipulation, as ozone can be easily mixed with the inlet gas flow.

Oxidative stress such as ozonation is known to reduce diversity and induce strong bactericidal effects at higher dosages (e.g., 5000–20,000 mg/m^3^), but our results showed the opposite, possibly due to a low-enough dosage to allow the subsistence of strains more sensitive to oxidative stress while stimulating the more robust strains, resulting in a more diverse microbiome [12, 13]. Given the diverse nature of xenobiotic degradation pathways, and the complexity of pollutant composition frequently seen in full-scale applications, ozonation is a possible method for the steady stimulation of microbiome diversity. Microbiome diversity has been reported to correlate directly with community stability and resistance to shocks, which are most commonly seen as spikes in loading rates during periods spanning from hours to months in full-scale operations depending on the particular scenario [36, 37]. The microbial co-occurrence network is a common modeling approach for describing characteristics and patterns of microbial co-occurrences and indicating ecological processes governing community structures [38]. The co-occurrence networks shown in Fig. 6 also support the hypothesis that the ozonated microbiome may be more resilient to shocks with a more decentralized microbiome network after ozone injection, resulting in a community structure less likely to experience changes as radical as there would be in the more centralized network of the control biofilter microbiome.

After ozonation, a series of microbes in the phylum *Proteobacteria* increased in relative abundance, most prominently including genera *Dokdonella* and *Mesorhizobium. Dokdonella* was identified as a major effective degrader of styrene in biofilters [39]. *Mesorhizobium* is a genus containing species strongly correlated to nitrogen fixation and denitrification with aerobic respiration, yet the genus was uncommonly reported to be associated with gaseous biofilters; correlation analysis of *Mesorhizobium*’s relative abundance and performance as seen in Fig. 5a shows that the genus strongly correlates with mineralization rate of the biofilter after ozonation; this could be explained by enhanced effective use of nitrogen sources through nutrient spraying [40]. *Proteobacteria* in general has been widely reported to contain dominant degraders of xenobiotics in both biofilter and wastewater treatments [41, 42]. *Proteobacteria* has also been reported to have a higher presence in the treatment of more complex pollutants and higher loading stress, inducing higher functional diversity [43]. A full list of families and genera that changed significantly with ozonation identified by LEfSe analysis can be seen in Supplementary Material 5. The degradation of xenobiotics such as toluene had highly diverse prokaryotic pathways, and different degrading communities have different abilities and propensity for metabolizing different parts of the pathway before final mineralization of VOCs [44]. A more even and less specialized phenotypic functional profile may be desirable in full-scale applications when the composition of pollutants fluctuates widely and contains a variety of VOCs groups, though this decreased metabolization rate of complex carbohydrate degradation and increases in other compounds caused by ozonation did not impinge the removal efficiency of the biofilter.

An increase in the stress tolerance of the microbiome is highly desirable in biofilters because common applications of biofilters include the emission of periodic fermentation, and highly diverse pollutants are produced by the chemical engineering and pharmaceutical industries depending on the production agenda. Such variations in the inlet introduce shocks and hinder the performance of biofilters by eliminating degrading strains unfit for the new environment [45]. The consistently increased stress-tolerant strains after ozonation will maintain performance and could mitigate such shocks in full-scale situations to an extent, a highly desirable outcome. An increase in the biofilm-forming population could be an issue in traditional biofilters with clogging issues, but the ozonation of biofilters was shown to greatly reduce the growth rate of overall biomass and alleviate pressure drop. The increase in the biofilm-forming population should therefore not be considered a hindrance to the steady operation of biofilters after the inoculation phase.

Functional predictions on the pathway level provided us with microscopic views of the microbiome function. Attesting to predicted phenotypic traits, increases in pathways responsible for cell membrane synthesis as a response to oxidative stress corresponded with shifts in different parts of toluene degradation pathways. Purine ribonucleoside degradation feeding the urea cycle along with aspartate degradation and pyrimidine deoxyribonucleoside degradation all contributed to the degradation of amino acids, indicating increased metabolic rates for carbon sources such as amino acids. Although not a targeted substrate for the microbiome in this study, organic acids are seen as toxic metabolites that build up in biofilters during long-term operation, and periodic neutralization is required to maintain the health of the degrading community [44]. Increases in metabolic activity indicated by enrichment in acid-degrading pathways are an advantage for biofilters with regards to lessening organic acid buildups.

Correlation analysis of performance and microbes showed that the commonly seen genus *Rhodococcus* negatively correlated with performance after ozonation. As numerous studies have reported that this genus is dominant in the degradation of xenobiotics in biofilter systems [41, 46], this indicated a shift in degrading contribution away from *Rhodococcus* and more toward ASVs from the genera *Devosia*, *Aquamicrobium*, and *Rhizobiales*, which had high positive correlation with performance. The genus *Pandoraea* was reported to be highly enriched in species capable of effective degradation of xenobiotics [47] and was positively correlated with *r* > 0.7 in both biofilters as shown in Fig. 6. Similar trends in these three genera were seen at the combined genus level.

The ozonation of biofilters has been shown to alleviate clogging issues while maintaining performance. This study revealed other potential improvements to the microbiome due to ozonation such as higher biodiversity and functional stability, attested by a more connected and robust topological network with less centralized distribution and lower average path, and a higher percentage of the microbiome being stress tolerant while phenotypically achieving higher metabolization rates for a variety of carbon sources. These factors all contribute to shaping a more robust and shock-resistant microbiome. Improving the microbiome is another positive aspect of ozone application along with the ease of retrofitting and solving clogging issues in full-scale applications.

## Conclusion

Shifts in major degrading species corresponding to performance, as well as increases in community biodiversity, could explain the consistent performances commonly seen in ozonation of biofilters despite the decrease in biomass. Increased metabolic activities of the ozonated microbiome for organic acids could ameliorate toxic accumulation issues present in long-term biofilter operation. The increased presence of stress-tolerant microbes as well as results from co-occurrence networks suggests that ozonation could not only mitigate clogging issues but also provide a microbiome more robust to loading shocks, which is desirable in full-scale biofilters. Additionally, functional predictions showed shifted pathway inclinations toward different parts of the toluene degradation pathway, and increased cell-membrane synthesis after ozonation. This study revealed numerous characteristics induced by ozone on the biofilter microbiome that explain the overall improvements in performances.

## Supplementary Information

**Additional file 1: Supplementary material 1. Fig. 1.** Alpha diversity indices of the samples from all sampling date. **Supplementary material 1. Fig. 2.** NMDS analysis of weighted-unifrac distance of all samples from all dates.

**Additional file 2: Supp. figure 5a.** Microbial community structure at phylum level, phyla of top 20 relative abundance are shown. **Supp. figure 5b.** Microbial community structure at genus level, genera of top 30 relative abundance are shown, others are combined and shown as “others”.

**Additional file 3.**

**Additional file 4.**

**Additional file 5.**

## Data Availability

Sequence data of all samples are available at GenBank BioProject accession number PRJNA656689. Codes used for statistical analysis and plotting can be accessed at https://github.com/myhyeung/Low-Dose-Ozonation-in-Gaseous-VOCs-Biofilter-Promotes-Community-Diversity-and-Robustness.
