# Supplementary material for: Low-dosage ozonation in gas-phase biofilter promotes community diversity and robustness
Source: Microbiome. 2021 Jan 12;9:14. doi: 10.1186/s40168-020-00944-4 (PMC7805145; doi:10.1186/s40168-020-00944-4)
Supplement: Supplementary file 2 — Additional file 1: Supplementary material 1. Fig. 1. Alpha diversity indices of the samples from all sampling date. Supplementary material 1. Fig. 2. NMDS analysis of weighted-unifrac distance of all samples from all dates. [file 40168_2020_944_MOESM1_ESM.docx]

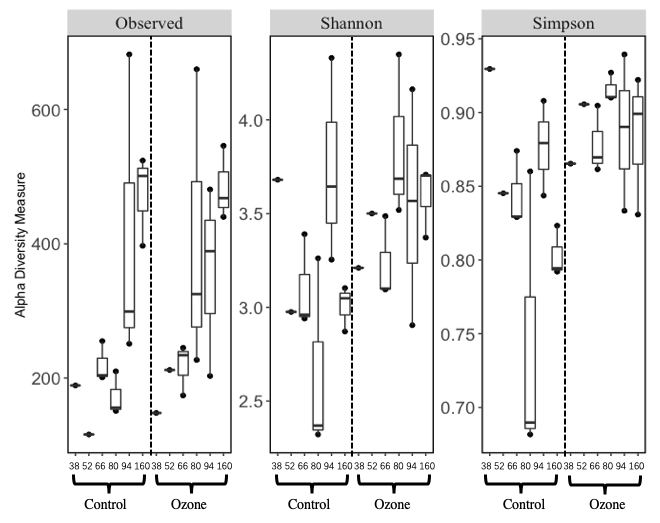


Supplementary material 1. Fig. 1. Alpha diversity indices of the samples from all sampling date.


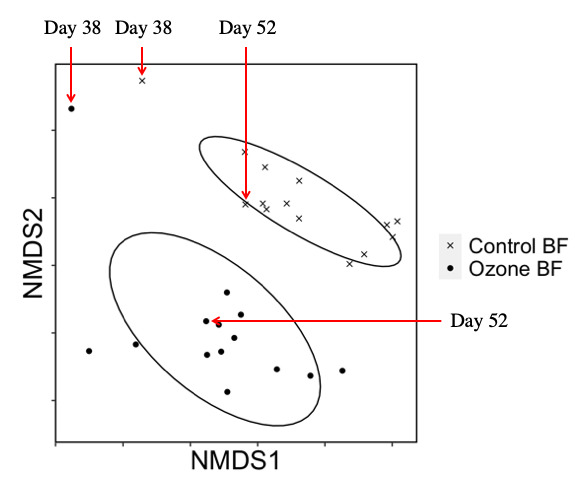


Supplementary material 1. Fig. 2. NMDS analysis of weighted-unifrac distance of all samples from all dates.
