# Supplementary material for: Low-dosage ozonation in gas-phase biofilter promotes community diversity and robustness
Source: Microbiome. 2021 Jan 12;9:14. doi: 10.1186/s40168-020-00944-4 (PMC7805145; doi:10.1186/s40168-020-00944-4)
Supplement: Supplementary file 3 — Additional file 2: Supp. figure 5a. Microbial community structure at phylum level, phyla of top 20 relative abundance are shown. Supp. figure 5b. Microbial community structure at genus level, genera of top 30 relative abundance are shown, others are combined and shown as “others”. [file 40168_2020_944_MOESM2_ESM.docx]

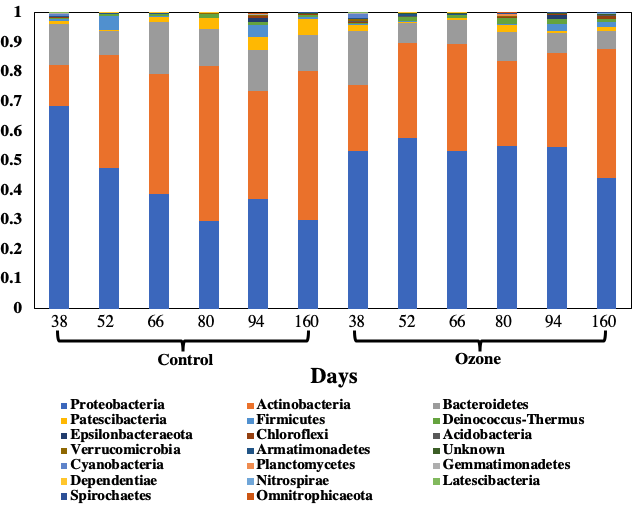


Supp. figure 5a. Microbial community structure at phylum level, phyla of top 20 relative abundance are shown.


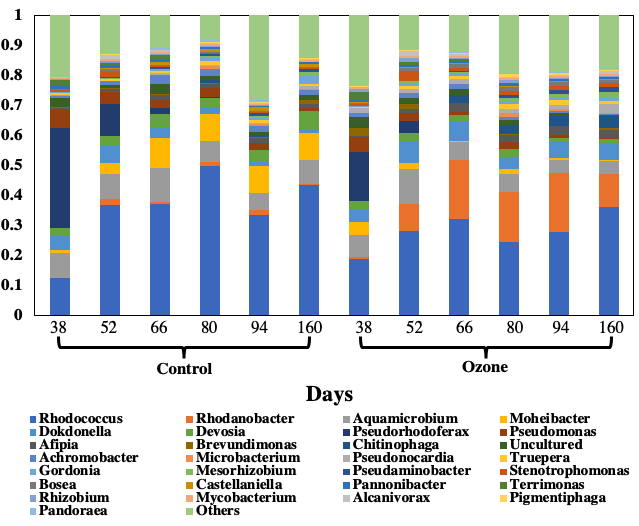


Supp. figure 5b. Microbial community structure at genus level, genera of top 30 relative abundance are shown, others are combined and shown as “others”.
